# Bioinformatics Screening of Tumor-Derived Neuropeptides Mediating Neuroimmune Axis of Head and Neck Cancer

**DOI:** 10.3390/cancers17152464

**Published:** 2025-07-25

**Authors:** Ravi Kishan, Gao Zhang, Weifa Yang, Yuxiong Su

**Affiliations:** 1 Division of Oral and Maxillofacial Surgery, Faculty of Dentistry, The University of Hong Kong, Hong Kong SAR, China; rkishan@connect.hku.hk; 2 Applied Oral Sciences & Community Dental Care, Faculty of Dentistry, The University of Hong Kong, Hong Kong SAR, China; gaozhang@hku.hk

**Keywords:** head and neck cancer, cancer neuroscience, neuropeptides, neuroimmune, immune checkpoint

## Abstract

**Background/Objectives:** Emerging studies have indicated the importance of intra-tumoral neuronal signals in tumor progression and immune modulation. However, there is limited insight into neuroimmune crosstalk, and the molecules involved are largely unknown. This study investigates the relationship between tumor-derived neuropeptides and immune modulation in head and neck squamous cell carcinoma (HNSC). **Methods:** By utilizing neuropeptide databases and web tools leveraging TCGA data, neuropeptides’ expression and their associations with neurotrophic factors, immune cell infiltration, and immune checkpoints were analyzed, followed by survival analysis. **Results:** Over half of the neuropeptides were expressed in HNSC, with 16% exhibiting differential expression compared to normal counterparts. Notably, differentially expressed neuropeptides showed significant correlations with neurotrophic factors, immune cell infiltration, and checkpoint genes. Further, their expression was significantly different in responder and non-responder patient samples subjected to immune checkpoint therapy. Neuropeptide genes—PTHLH, NMB, GAST, APLN, and LYNX1—were identified and emerged as crucial mediators in neuroimmune crosstalk. Additionally, the neurotrophic gene NTRK1 exhibited extensive correlation with immune checkpoint genes, underscoring the prevalence of neuroimmune crosstalk in HNSC. **Conclusions:** These findings shed light on the role of tumor-derived neuropeptides in neuroimmune regulation in HNSC, offering valuable insights for future studies to decode the cancer neuroscience of HNSC progression and therapy.

## Simple Summary

Head and neck cancers advance rapidly by establishing dense nerve networks that not only promote tumor growth but also enable immune evasion. Despite their critical role, the specific molecules driving this nerve–immune communication—and representing promising therapeutic targets—remain largely unidentified. In this study, we hypothesized that tumor-secreted neuropeptides act as pivotal mediators within the neuroimmune axis, orchestrating interactions that facilitate cancer progression and treatment resistance. Through comprehensive analysis of existing data, we identified key neuropeptides strongly associated with factors driving nerve density, suppressed immune activity, and resistance to therapy. These molecules represent promising, targetable “messengers” that link neural and immune pathways within the tumor microenvironment. Our findings lay a crucial foundation for future mechanistic research aimed at disrupting nerve–immune crosstalk, opening new avenues for innovative therapies to improve outcomes for patients battling head and neck cancers.

## 1. Introduction

Head and neck squamous cell carcinoma (HNSC) is a highly aggressive malignancy characterized by dense innervation and frequent perineural invasion (PNI), a process observed in up to 80% of cases and associated with poor prognosis, recurrence, and treatment resistance [1,2]. PNI, where cancer cells invade and spread along nerves, is a hallmark of HNSC aggressiveness. Beyond PNI, emerging evidence highlights a bidirectional crosstalk between tumors and nerves, creating a dynamic microenvironment that fuels cancer progression and immune suppression. On one hand, tumors actively recruit neurons into the tumor microenvironment (TME) by mediating neurogenesis, axonogenesis, and neuronal reprogramming to facilitate growth and dissemination. On the other hand, peripheral nerves within the TME secrete neurotransmitters, neuropeptides, and growth factors that directly stimulate tumor proliferation, angiogenesis, and immune evasion [3,4,5]. This neuro–tumoral symbiosis establishes a highly immunosuppressive TME, marked by dysfunctional immune cell infiltration, upregulation of immune checkpoints, and resistance to conventional therapies [6,7]. These findings evidence that neuropeptides and neurotransmitters are crucial for mediating the pro-tumor microenvironment, including immune suppression conducive for tumor growth. However, what triggers a tumor to initiate neuron infiltration and immune suppression at the onset of tumor inception remains poorly understood. Furthermore, the molecular mediators of neuroimmune crosstalk in HNSC are largely unexplored.

Central to this interplay are tumor-derived neuropeptides, which have been linked to driving tumor innervation, angiogenesis, and immune suppression. Neuropeptides are small protein-like molecules that are typically secreted by neurons. Interestingly, various solid tumors, including breast, lung, and pancreatic cancers, ectopically express neuropeptides such as Neuropeptide Y (NPY), Substance P (SP), and Vasoactive Intestinal Peptide (VIP), mediating communication between neurons, immune cells, and cancer cells [8,9,10]. They signal through G-protein-coupled receptors (GPCRs) to regulate diverse physiological processes, including neurogenesis, immune modulation, and metabolic reprogramming [11,12]. These tumor-derived neuropeptides can influence nerve responses to neurotransmitters and interact with TME components, including immune and endothelial cells, contributing to neuroendocrine, metabolic, and immunosuppressive changes. For instance, NPY promotes breast cancer metastasis by stimulating endothelial cell migration via Y2 receptors, while SP enhances pancreatic cancer proliferation by activating neurokinin-1 receptor (NK1R)-mediated ERK signaling. Further, NPY has also been shown to promote M2 macrophage polarization and regulatory T-cell (Treg) infiltration, creating an immunosuppressive niche [13,14,15,16].

In addition to their effects on immune cells, tumor-derived neuropeptides interact with neurotrophic factors such as Nerve Growth Factor (NGF) and Brain-Derived Neurotrophic Factor (BDNF), which are critical for axonogenesis and nerve recruitment inside the TME. Neuropeptides like NPY and SP can amplify the activity of these factors, increasing nerve density and promoting cancer cell survival and drug resistance [17,18]. This dual role—modulating both immune evasion and neuronal plasticity—positions tumor-derived neuropeptides as master regulators of the TME.

In HNSC, tumor-derived neuropeptides could be hypothesized to play a similar role, acting as critical mediators of the neuroimmune axis by influencing both neuronal remodeling and immune responses, thereby fostering pro-tumor cues within the TME. The neuroimmune axis in HNSC represents a complex network of interactions between neurons, immune cells, and cancer cells, orchestrated by signaling molecules such as neurotransmitters, neuropeptides, and cytokines. Tumor-derived neuropeptides are emerging as key players in this axis, modulating both neuronal plasticity and immune responses. Despite their multifaceted roles, the contributions of tumor-derived neuropeptides to immune checkpoint regulation, immune cell infiltration, and neuronal remodeling in HNSC have not been systematically investigated. Therefore, the objective of this study is to investigate the comprehensive association of tumor-derived neuropeptides with neurotrophic factors, immune checkpoints, and immune infiltration in the TME of HNSC. The comprehensive analysis provides a foundation for identifying neuronal markers that could be targeted to modulate the TME and tumor prognosis.

## 2. Methods

### 2.1. Acquisition of Neuropeptide Dataset

The neuropeptide datasets for this study were extracted from the online neuropeptide repository (http://www.neuropeptides.nl/; accessed on 10 November 2024). This repository contains the neuropeptide gene, gene symbol, family, precursor, and active peptide lists. Further, the extracted neuropeptide list was validated with other neuropeptide databases: NeuroPedia (http://proteomics.ucsd.edu/Software/NeuroPedia/; accessed 11 November 2024) and NeuroPep (http://isyslab.info/NeuroPep/; accessed on 11 November 2024) [19,20]. All entries are manually validated and annotated with information such as source organisms, tissue specificity, families, names, post-translational modifications, 3D structures (if available), and references to the literature. The constructed neuropeptide dataset can be found in Supplementary Table S1.

### 2.2. Differential Gene Expression Analysis

The differential gene expression and fold change analysis of the neuropeptide genes in the normal and tumor samples along with stage plots was performed using GEPIA2 (Gene Expression Profiling Interactive Analysis) (http://gepia2.cancer-pku.cn/; accessed on 10 March 2025), an advanced web server analysis tool for the RNA expression data of normal and tumor samples based on The Cancer Genome Atlas (TCGA) and the Genotype-Tissue Expression (GTEx) datasets [21]. The platform employs novel gene signature quantification methodologies inspired by single-cell sequencing investigations and allows users to upload and compare their own RNA-seq data to the TCGA and GTEx datasets. Our study utilized bulk mRNA sequencing data for gene expression analysis in tumor tissues. Although this approach does not resolve cell type-specific expression within the heterogeneous tumor microenvironment, it provides a comprehensive overview of the overall gene expression changes associated with tumorigenesis. This broad perspective is crucial for identifying key molecular players and potential biomarkers, serving as an essential foundation for further targeted investigations. For the determination of fold change value, the median mRNA expression value was extracted in both the tumor and normal samples, and fold change was calculated by the following formula: Fold change = log_2_ (Tumor) − log_2_(Normal). Unless stated, the *p*-value of each analysis is *p* < 0.05.

### 2.3. Gene Pair Correlation and Immune Cell Infiltration Analysis

The gene pair correlation and immune cell infiltration analysis with respect to all neuropeptide genes was analyzed via the Tumor Immune Estimation Resource (TIMER)—a web server tool that provides pre-calculated levels of six immune subgroups infiltrating tumors in 10,897 samples from 32 different forms of cancer (https://cistrome.shinyapps.io/timer/; accessed on 14 March 2025). It features six analytic modules investigating relationships between immune infiltrates and various parameters, including gene expression, somatic mutations, somatic copy number variations, and clinical outcomes, as well as the correlation between user-defined gene pairs across different cancer types [22].

### 2.4. Immunotherapy Outcome

To analyze the impact of neuropeptide genes in immune checkpoint blockade (ICB) therapy, the Immune Checkpoint Blockade Therapy Atlas (ICBatlas) was used (http://bioinfo.life.hust.edu.cn/ICBatlas/; accessed on 20 March 2025). This online resource, which includes transcriptome and clinical datasets from 1515 patients treated with ICB therapy across nine cancer types, allowed us to examine neuropeptide gene expression in both the responder and non-responder samples [23]. The clinical information analysis categorized the samples into responder/non-responder or pre-treatment/on-treatment groups, followed by differential gene expression and *p*-value analysis.

### 2.5. Survival Analysis

The overall survival association with differentially expressed neuropeptide genes in HNSC patients was analyzed using the Kaplan–Meier Plotter (www.kmplot.com; accessed on 13 April 2025). It is a tool to generate survival plots based on the gene expression data and survival information obtained from the GEO, EGA, and TCGA cancer datasets. This tool segregates the patient samples into a high and low median expression of the genes based on the hazard ratio (HR), 95% confidence intervals (CIs), and log-rank *p*-value to estimate the OS, FP, and PPS of the patients in various cancer types.

## 3. Results

### 3.1. More Than Half of Neuropeptide Genes Expressed in HNSCC

**Figure 1:**
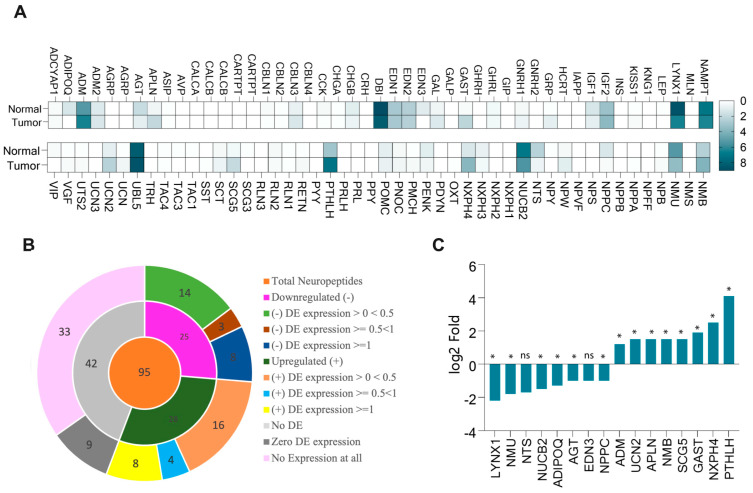
Expression of neuropeptides in normal cells and HNSC. (**A**) mRNA expression of neuropeptides in normal and tumor (HNSC) cells. (**B**) The core of the doughnut chart shows the total number of significant neuropeptide genes found in various databases. The first layer of the chart shows the number of upregulated and downregulated genes in HNSC and the genes showing no differential expression (DE). The second layer shows the log2 fold change data of the gene expression in HNSC as compared to normal cells. (**C**) Individual log2 fold change data of the upregulated and downregulated genes having DE or log2 fold change > = 1 (* *p* < 0.05; ns: no statistical significance).

The mRNA expression analysis of the neuropeptides revealed that 56% of the neuropeptide genes were differentially expressed in HNSC (Figure 1A,B). Among them, approximately 29.5% were upregulated, while 26.5% were downregulated. We further conducted differential expression (DE) analysis, which indicated that approximately 16% of the neuropeptide genes had a fold change greater than 1log2 (8% upregulated and 8% downregulated). Notably, the most significantly upregulated gene was *Parathyroid Hormone-Like Hormone* (*PTHLH*) (log_2_ fold change value = 4.1; *p* < 0.05), while *Ly6/Neurotoxin 1* (*LYNX1*) showed the most significant downregulation (log_2_ fold change value = −2.2; *p* < 0.05) (Figure 1C). The expression data of all differentially expressed neuropeptide genes (log2-scale) is depicted in Supplementary Figure S1.

### 3.2. PTHLH, NMB, GAST, LYNX1, and AGT Neuropeptides Linked to Immune Cell Infiltration in HNSC

**Figure 2:**
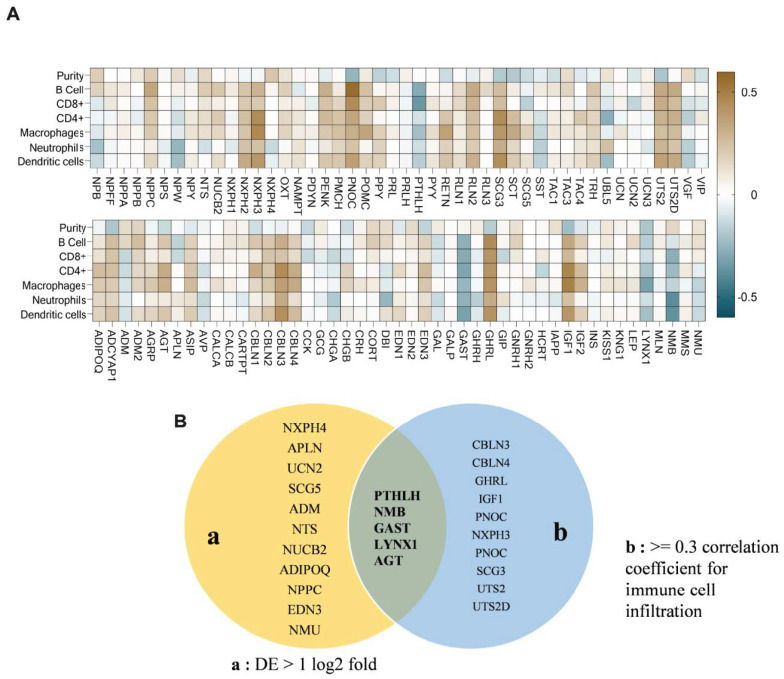
Correlation of neuropeptides with immune cell infiltration. (**A**) Immune cell infiltration correlation data for neuropeptides. (**B**) Venn diagram showing neuropeptides having significant DE and immune cell infiltration levels (*p* < 0.05).

Approximately 15% of the neuropeptides were associated with at least one of the six immune cell types analyzed, including B cells, CD8^+^, CD4^+^, macrophages, neutrophils, and dendritic cells. Among them, *Cerebellin 3 Precursor* (*CBLN3*), *Ghrelin And Obestatin Prepropeptide* (*GHRL*), *Insulin-like Growth Factor-1* (*IGF1*), and *Prepronociceptin* (*PNOC*) exhibited the highest positive correlations (correlation value > 0.4; *p* < 0.05) with immune cell infiltration (Figure 2A). Intriguingly, despite these strong associations, these neuropeptides did not exhibit differential expression in HNSC. Conversely, neuropeptides *PTHLH* (with B cells and CD8+) and *Neuromedin B* (*NMB*; with neutrophils and dendritic cells) showed negative correlations with immune cell infiltration, which demonstrated differential expression in HNSC. This duality prompted further investigation. After scrutinizing their fold change values and considering their significance in immune cell infiltrations, we identified a subset of five neuropeptides that overlapped, including *PTHLH*, *NMB*, *Gastrin* (*GAST*), *LYNX1*, and *AGT* (Figure 2B). A detailed view of the correlation data for these neuropeptides is presented in Supplementary Figure S2.

### 3.3. PTHLH, SCG5, APLN, and UCN2 Correlated with Immune Checkpoint Genes

**Figure 3:**
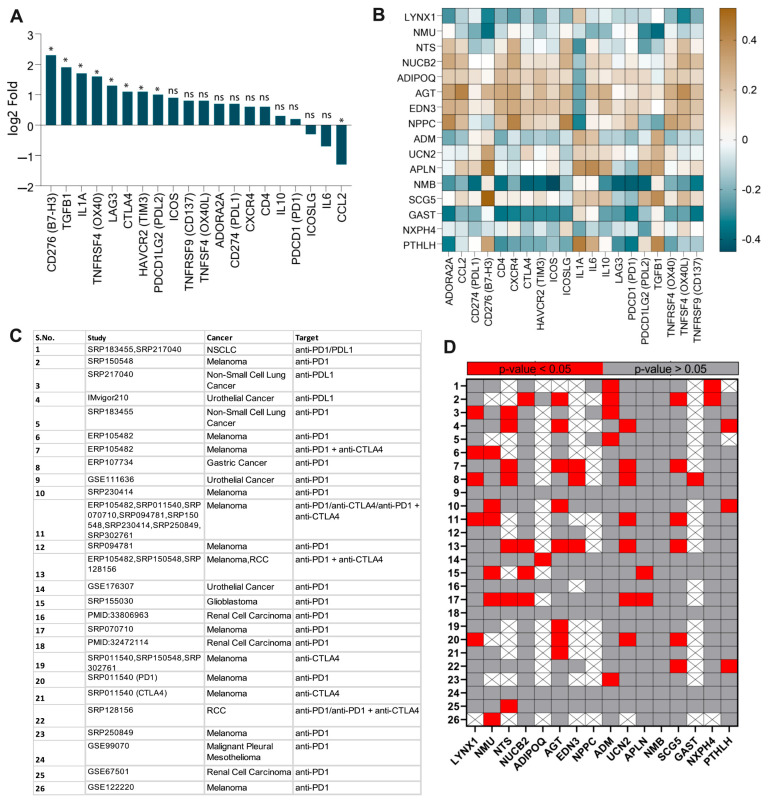
Association of neuropeptides with immune checkpoints. (**A**) Differential expression (log2-fold change values) of immune checkpoint genes (ICGs) in HNSC. (**B**) Correlation of differentially expressed (DE) neuropeptide genes with targetable immune checkpoint genes. (**C**) Datasets of immunotherapy conducted on patients in various studies. (**D**) *p*-values of each neuropeptide gene expression in responder and non-responder samples subjected to immunotherapy (* *p* < 0.05; ns: no statistical significance).

Because of the important role of immune checkpoint genes (ICGs) in regulating immunomodulation, we analyzed the HNSC-specific expression patterns of each ICG. Our analysis revealed that *B7 Homolog 3* (*CD276/B7-H3*) (log_2_fold change: 2.3; *p* < 0.05), *Transforming Growth Factor Beta-1* (*TGFB1*) (1.9; *p* < 0.05), *Interleukin 1-Alpha* (*IL1A;* 1.7; *p* < 0.05), *Tumor Necrosis Factor Receptor Superfamily*, *member 4* (*TNFRSF4/OX40*; 1.6; *p* < 0.05), *Lymphocyte Activation Gene 3* (*LAG3*; 1.3; *p* < 0.05), *Cytotoxic T-Lymphocyte Associated Protein 4* (*CTLA4*; 1.1; *p* < 0.05), *T-cell Immunoglobulin domain and mucin domain 3* (*TIM3*; 1.1; *p* < 0.05), and *Programmed Cell Death 1 Ligand 2* (*PDL2*; 1.0; *p* < 0.05) were the highly upregulated ICGs in HNSC as compared to their normal counterparts. While *TNFSF4/OX40L* (0.8), *Programmed Cell Death 1 Ligand 1* (*PD-L1*; 0.7), and *Programmed Cell Death 1* (*PD-1*; 0.2) showed moderate upregulation, the difference was not statistically significant (ns). In contrast, *Inducible T Cell Costimulator Ligand* (*ICOSLG*; −0.3; ns), *Interleukin 6* (*IL6*; −0.7; ns), and *C-C Motif Chemokine Ligand 2* (*CCL2*; −1.3, *p* < 0.05) were downregulated (Figure 3A). Collectively, these findings highlight the distinct expression dynamics of ICGs in HNSC, suggestive of their relevance in HNSC immunomodulation. The box plot for the ICG expression is presented in Supplementary Figure S3.

The correlation analysis between neuropeptides and ICGs, summarized in Table 1, revealed intricate regulatory associations. Consistent with expectations, upregulated neuropeptides, such as *Secretogranin V* (*SCG5*), *APLN*, and *Urocortin-2* (*UCN2*), were positively correlated with ICGs, whereas downregulated neuropeptide genes *Neuromedin U* (*NMU*) and *LYNX1* were negatively correlated with ICGs (*p* < 0.05), indicating they could be involved in immunomodulation. In contrast, upregulated neuropeptides *GAST* and *NMB* correlated negatively with upregulated ICGs (*p* < 0.05), which is indicative of their potential role in anti-tumor immunomodulation (Figure 3B).

Further, the analysis of patient datasets from 26 relevant studies revealed significant differences in neuropeptide expression between responder and non-responder samples derived from cancer patients treated with cancer immunotherapy (Figure 3C). Except for studies 9, 18, and 24, the analysis of the data derived from most of the studies demonstrated statistical significance (*p* < 0.05) for neuropeptides such as *LYNX1*, *NMU*, *NTS*, *AGT*, *ADM*, *UCN2*, and *SCG5* (Figure 3D). Notably, *LYNX1*, *SCG5*, and *UCN2*, which were correlated with ICGs in earlier analyses, showed consistent significance, highlighting their potential as key modulators of immunotherapeutic efficacy.

### 3.4. Differentially Expressed Neuropeptides Correlated with Key Neurotrophic Factors (ARTN, TGFB1, SEMA4F) in HNSC

**Figure 4:**
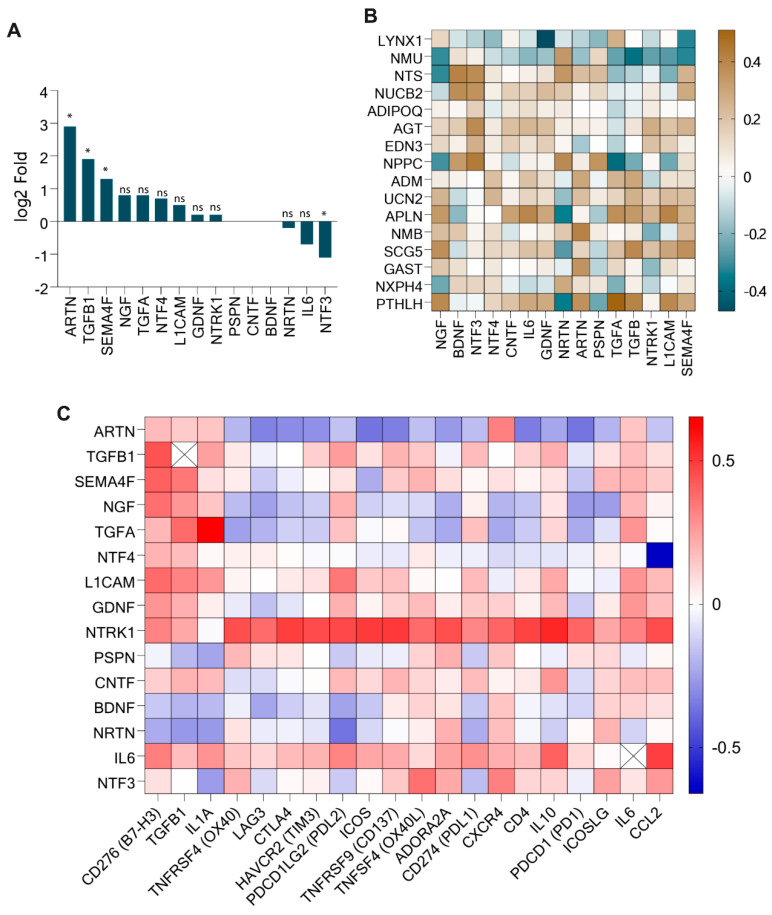
(**A**) Differential expression (log2-fold change value) of neurotrophic factor genes in HNSC (* *p* < 0.05; ns: no statistical significance). (**B**) Correlation map of neuropeptides and neurotrophic genes in HNSC. (**C**) Correlation map of neurotrophic factor and immune checkpoint genes (*p* < 0.05).

Neurotrophic factors, or neurotrophins, are a family of growth factors responsible for the growth, development, maintenance, and differentiation of both the central and peripheral nervous systems [24]. Considering their importance for neuron survival, we identified neurotrophic factor genes by conducting a literature review and evaluated their expression in HNSC. Our analysis identified differentially expressed neurotrophic factor genes in HNSC compared to normal tissue, and the differences were statistically significant (Figure 4A). Specifically, *Artemin* (*ARTN*; log_2_ fold change: 2.9; *p* < 0.05), *TGFB1* (1.9; *p* < 0.05), and *Semaphorin-4F* (*SEMA4*; 1.3; *p* < 0.05) exhibited the highest levels of upregulation. Conversely, *Neurturin* (*NRTN*; −0.2; ns), *Interleukin-6* (*IL6*; −0.7; ns), and *Neurotrophin-3* (*NTF3*; −1.1; *p* < 0.05) were downregulated, while *Nerve Growth Factor* (*NGF*; 0.8; ns), *Neurotrophin-4* (*NTF4*; 0.7; ns), *Glial Cell Line-Derived Neurotrophic Factor* (*GDNF*; 0.2; ns), and *Neurotrophic Receptor Tyrosine Kinase-1* (*NTRK1*; 0.2; ns) showed minimal upregulation. Other neurotrophic factors, including *Persephin* (*PSPN*), *Ciliary Neurotrophic Factor* (*CNTF*), and *Brain-Derived Neurotrophic Factor* (*BDNF*), exhibited no differential expression. These findings suggest a prominent role for *ARTN*, *TGFB1*, and *SEMA4F* in HNSC, while other neurotrophic factors may play less critical roles in this context. The box plot for the neurotrophic factor gene expression is presented in Supplementary Figure S4.

The correlation analysis between neurotrophic factors and differentially expressed neuropeptide genes revealed distinct association patterns (*p* < 0.05; Figure 4B and Table 2). Upregulated neuropeptide genes, such as *PTHLH*, *Gastrin* (*GAST*), *Secretogranin V* (*SCG5*), *Neuromedin B* (*NMB*), and *Apelin* (*APLN*), were positively correlated with upregulated neurotrophic factors, which were consistent with their roles in tumor progression and neuroimmune crosstalk. However, *PTHLH* and *APLN* also demonstrated negative correlations with downregulated neurotrophic genes, indicating a complex regulatory mechanism. Downregulated neuropeptides, including *Natriuretic Peptide C* (*NPPC*) and *Neurotensin* (*NTS*), exhibited negative correlations with upregulated neurotrophic factors but positive correlations with downregulated or non-differentially expressed neurotrophic factors. Similarly, *Nucleobindin-2* (*NUCB2*) showed positive correlations with non-differentially expressed and downregulated neurotrophic genes, while *Neuromedin U* (*NMU*) and *LYNX1* displayed negative correlations with upregulated neurotrophic genes. Notably, *Angiotensinogen* (*AGT*) was positively associated with downregulated neurotrophic genes.

The intricate association between neurotrophic genes, known for their pivotal role in neuron growth regulation, and immune checkpoint genes (ICGs), essential for immunomodulation, was explored in this analysis (Figure 4C). The neurotrophic gene *NTRK1*, encoding tropomyosin receptor kinase (TRK), was found to positively correlate with multiple ICGs, including *CTLA4* (correlation value = 0.487), *OX40* (0.449), *LAG3* (0.378), *TIM3* (0.457), *PDL2* (0.461), *IL10* (0.545), *CD137* (0.497), and *CCL2* (0.452). Additionally, the immune checkpoint gene *CCL2* was correlated with neurotrophic genes *NTF4* (−0.660) and *IL6* (0.485), while the highly upregulated immune checkpoint gene *CD276/B7H3* showed positive correlations with upregulated neurotrophic genes *TGFB1* (0.440), *SEMA4F* (0.404), and *NGF* (0.369). The neurotrophic gene *ARTN* displayed negative correlations with several ICGs, such as *PD1* (−0.360), *CD137* (−0.336), *ICOS* (−0.363), *CTLA4* (−0.301), and *LAG3* (−0.327), but it showed a positive correlation with *CXCR4* (0.326). Moreover, *IL1A* was strongly correlated with *TGFA* (0.653), and a general pattern of neuropeptide correlations with neurotrophic factors and ICGs was observed, as highlighted in the previous analysis.

### 3.5. LYNX1, UCN2, AGT, SCG5, and GAST Correlated with HNSC Survival

**Figure 5:**
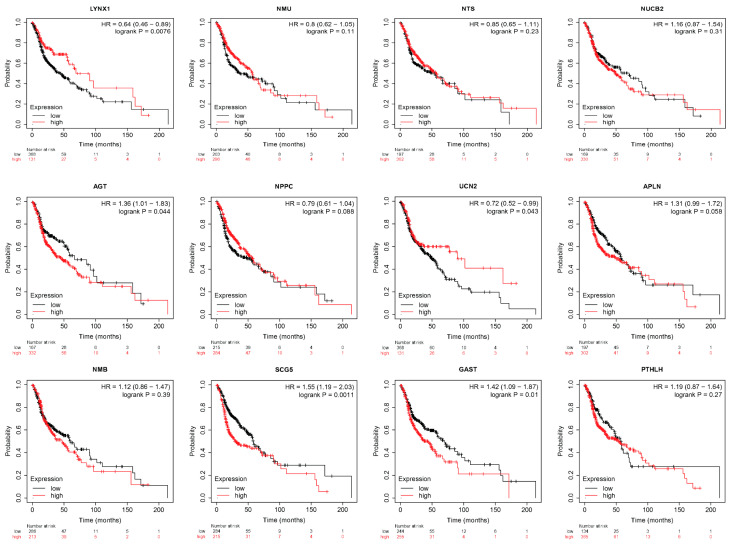
Kaplan–Meier plotter for the overall survival analysis of the neuropeptide expression.

Based on the analysis, 12 of the 16 differentially expressed neuropeptide genes were directly or indirectly correlated with neuroimmune factors in HNSC. The prognostic analysis of these genes using the Kaplan–Meier Plotter indicates that increased *LYNX1* (HR = 0.64, *p* = 0.0076), *UCN2* (HR = 0.72, *p* = 0.043), and decreased *AGT* (HR = 1.36, *p* = 0.044), *SCG5* (HR = 1.55, *p* = 0.0011), and *GAST* (HR = 1.42, *p* = 0.01) gene expressions (mRNA levels) were associated with longer overall survival rates (Figure 5). The prognostic value of neuropeptide genes was analogous to its correlation with immune checkpoint genes, such as downregulated gene *LYNX1*, which showed only a negative correlation with upregulated ICGs and demonstrated good prognostic value.

### 3.6. Stage Plot Analysis Revealed Consistent Expression of Neuropeptide Genes

**Figure 6:**
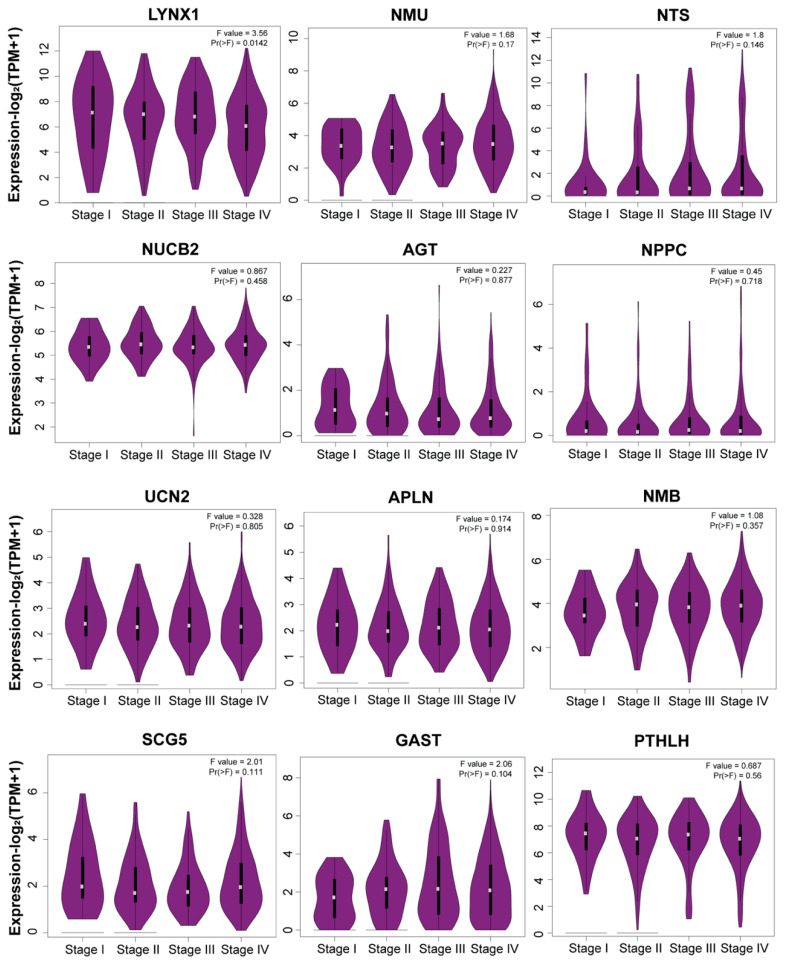
Stage plot analysis of neuropeptide genes correlated with neuroimmune factors in HNSC (http://gepia2.cancer-pku.cn/; accessed on 2 June 2025).

The analysis of the expression profiles of 12 neuroimmune-associated neuropeptide genes across tumor stages (Stages I–IV) revealed no significant differences in the expression levels of 11 genes (*p* > 0.05), although LYNX1 exhibited a significant downregulation in Stage I compared to later stages (*p* = 0.0142) (Figure 6). This observation suggests that these neuropeptide genes are dysregulated during tumor initiation to modulate the neuroimmune axis, ultimately paving the way for tumor formation; LYNX1 could have a protective role, and its expression decreased as the tumor progressed from Stage I to Stage IV. Notably, LYNX1 was downregulated in HNSC overall.

## 4. Discussion

This study provides compelling insights into the role of tumor-derived neuropeptides in mediating neuroimmune crosstalk in HNSC. By emphasizing the intricate interplay between neuropeptides, neurotrophic factors, and immune components, neuropeptides *PTHLH*, *NMB*, *APLN*, *GAST*, and *LYNX1* were found to be critical players.

More than half of the neuropeptides showed expression in HNSC, supporting the idea that HNSC tumor cells are active participants in neuropeptide secretion. Interestingly, widely studied neuropeptides like CGRP showed no differential expression, contrasting with other reports of CGRP upregulation in HNSC, suggesting neurons as the primary source of its secretion [8,25]. It is important to note that neuronal genetic material is typically confined to ganglionic regions away from the tumor site [26]. Hence, evaluating mRNA expression of tumors could not account for neuronal expression.

The infiltration of immune cells plays a critical role in determining the progression, elimination, and survival of tumors [27]. B cells, for example, can restrict tumor development by producing tumor-reactive antibodies, enhancing tumor elimination by NK cells and macrophage phagocytosis, and priming CD4+ and CD8+ T lymphocytes [28]. CD8+ T cells and activated memory CD4+ T cells are well-recognized indicators of an immunoactive environment and a better prognosis [29]. *PTHLH*, *NMB*, *GAST*, *LYNX1*, and *AGT* showed both differential expression and significant correlation with immune cells’ infiltration in HNSC. Further, the correlation of *PTHLH*, *SCG5*, *APLN*, and *UCN2* with ICGs, including *PD-1*, *CTLA-4*, and *B7H3*, highlights their potential to influence immune escape mechanisms. PD1/L1/L2 and CTLA4 have been a popular target for immune checkpoint therapy [30,31,32,33]. Recently, B7H3/CD276 gained much interest in cancer immunotherapy due to its significantly higher expression in tumors [34]. It was also the most upregulated ICG in HNSC. Additionally, survival analyses of the neuropeptides showed that their prognostic value was analogous to their correlations with ICGs, further reinforcing their impact on immunomodulation. These findings suggest that neuropeptides may act as indirect regulators of immune checkpoint pathways, shaping the immunosuppressive landscape of HNSC. Consequently, neuropeptide profiling could serve as a valuable approach to enhance immunotherapeutic strategies, particularly by predicting patients’ responses to immune checkpoint blockade (ICB) therapy. Targeting neuropeptides or their signaling pathways may modulate the tumor immune microenvironment, potentially overcoming resistance mechanisms and improving therapeutic outcomes. Future research should focus on integrating neuropeptide profiling into personalized treatment plans and exploring combination therapies that include neuropeptide modulation alongside immune checkpoint inhibitors, aiming to develop more effective and tailored immunotherapeutic interventions for HNSC.

Intra-tumoral nerve density has a multifaceted role in driving tumor aggressiveness. Apart from facilitating immunomodulation, a recent study highlights its role in eliciting tumor metabolic plasticity by providing mitochondria to malignant cells [35]. The association between neuropeptides and the most differentially expressed neurotrophic factors, such as *ARTN*, *TGFB1*, and *SEMA4F*, in HNSC further highlights their role in driving nerve density within the TME. ARTN is essential for sensory neuron survival, and it is highly relevant in the context of HNSC, where high sensory neuron innervation has been observed [36]. TGFB1 has diverse roles in tumor biology, including neuronal development and survival, while SEMA4F has been shown to regulate neurogenesis and axonogenesis in prostate cancer [37,38]. The strong correlations between neuropeptides *PTHLH*, *NMB*, *APLN*, and *GAST* with these neurotrophic factors suggest a coordinated mechanism through which tumor cells recruit and sustain neuronal infiltration [39,40,41]. Some of these neuropeptides have already been implicated in tumor aggressiveness and metastasis in various cancers [42,43,44,45,46]. However, its direct role in neuroimmune interactions remains underexplored. These findings provide a strong foundation for further research into the mechanisms underlying neuropeptide-mediated interactions in the TME and their potential as therapeutic targets in HNSC. Moreover, it also provides potential research directions to investigate and advance the field of cancer neuroscience, which currently lacks fundamental knowledge to translate into clinical applications [47].

## 5. Conclusions

Our study provides an initial overview of tumor-derived neuropeptides that could be involved in neuroimmune modulation in HNSC. The majority of the neuropeptide genes were aberrantly expressed in HNSC tumors, indicating these neuropeptides have diverse roles in HNSC carcinogenesis. Most differentially expressed neuropeptide genes were significantly correlated with neurotrophic factors and immune checkpoint genes. This highlights the interconnectedness between neuropeptides, neurotrophic factors, and immune checkpoint regulation in HNSC. Since neuroimmune crosstalk in cancer is very new and emerging, further investigation and extensive study of these neuropeptides could yield novel insights into the progression of HNSC. By unraveling the intricate interactions between neuropeptides, neurotrophic factors, and immune checkpoints, potential therapeutic targets may be identified, leading to the development of innovative treatment strategies for HNSC.

## Figures and Tables

**Table 1:** Summary of correlation between neuropeptide and immune checkpoint genes.

Neuropeptide Genes		Upregulated Checkpoint Gene (Correlation Value)	Downregulated Immune Checkpoint Gene (Correlation Value)
Upregulated	***PTHLH***	CD276/B7H3 (0.322), TGFB1 (0.407), IL1A (0.44), ADORA21 (−0.331), PD1 (−0.345)	
	***GAST***	CTLA4 (−0.304), TIM3 (−0.305), ICOS (−0.304), CD137 (−0.349), ADORA2A (−0.349), CXCR4 (−0.324), CD4 (−0.354), PD1 (−0.342)	
	***SCG5***	B7H3 (0.528), TGFB1 (0.390)	
	***NMB***	LAG3 (−0.396), CTLA4 (−0.397), TIM3 (−0.378), PDL2 (−0.392), ICOS (−0.449), CD137 (−0.341), PDL1 (−0.372), CD4 (−0.365), PD1 (−0.403)	
	***APLN***	B7H3 (0.466), TGFB1 (0.337), IL1A (0.368), PDL2 (0.318)	IL6 (0.396)
	***UCN2***	B7H3 (0.323)	
Downregulated	***NPPC***	OX40 (0.327), ADORA2A (0.398), CXCR4 (0.425) IL1A (−0.317)	ICOSLG (0.413)
	***AGT***	OX40L (0.387), ADORA2A (0.325), CXCR4 (0.321),	CCL2 (0.4240)
	***NUCB2***	CXCR4 (0.309), ADORA2A (0.3)	
	***NMU***	B7H3 (−0.364), TGFB1 (−0.375), PDL2 (−0.3)	
	***LYNX1***	B7H3 (−0.319), OX40L (−0.319)	

**Table 2:** Summary of correlation between neuropeptides and neurotrophic genes.

Neuropeptide Genes		Upregulated Neurotrophic Genes (Correlation Value)	Non-Differentially Expressed Neurotrophic Genes (Correlation Value)	Downregulated Neurotrophic Genes (Correlation Value)
**Upregulated**	***PTHLH***	ARTN (0.362), NGF (0.388), TGFB (0.407), TGFA (0.510), L1CAM (0.390)		NRTN (−0.348)
	***GAST***	ARTN (0.356)		
	***SCG5***	TGFB (0.390), NGF (0.370), SEMA4F (0.366), L1CAM (0.3)		
	***NMB***	ARTN (0.410)		
	***APLN***	TGFB (0.337), NGF (0.338), TGFA (0.370), L1CAM (0.405)		NRTN (−0.396)
**Downregulated**	***NPPC***	TGFA (−0.388)	PSPN (0.353), BDNF (0.331)	NRTN (0.387), NTF3 (0.434)
	***AGT***			NTF3 (0.382)
	***NUCB2***		BDNF (0.377)	NTF3 (0.361)
	***NTS***	NGF (−0.316)	BDNF (0.411)	NRTN (0.344), NTF3 (0.38)
	***NMU***	TGFB (−0.375), SEMA4F (−0.323), NGF (−0.310)		NRTN (0.339)
	***LYNX1***	SEMA4F (−0.327), GDNF (−0.470)		

## Data Availability

The data supporting this study are available from the corresponding authors upon reasonable request.

## Supplementary Materials

The following supporting information can be downloaded at https://www.mdpi.com/article/10.3390/cancers17152464/s1: Figure S1: The individual mRNA expression boxplot of the upregulated and downregulated neuropeptide genes (having log2 fold change >= 1), in HNSC and normal tissue in GPIA2 (http://gepia2.cancer-pku.cn/#analysis accessed on 10 March 2025; * *p* < 0.05); Figure S2: Correlation of immune cells infiltration level of the selected neuropeptides which also showed significant differential expression: PTHLH, GAST, NMB, AGT and LYNX1 (https://cistrome.shinyapps.io/timer/ accessed on 14 March 2025); Figure S3: Expression of immune checkpoint genes (ICGs) in HNSC (* *p* < 0.05). CD276, CTLA4, HAVCR2, LAG3, PDCDLG2, TGFB1 and TNFRSF4 showed significant upregulation in HNSC tumor as compared to normal tissue; while CCL2 was significantly downregulated; Figure S4: Expression of neurotrophic factors/neurotrophins in HNSC (* *p* < 0.05). In HNSC, neurotrophins gene-ARTN, SEMA4F and TGFB1 were significantly upregulated as compared to normal counterparts; Table S1. Neuropeptide List.
