# Supplementary material for: Bioinformatics Screening of Tumor-Derived Neuropeptides Mediating Neuroimmune Axis of Head and Neck Cancer
Source: Cancers (Basel). 2025 Jul 25;17(15):2464. doi: 10.3390/cancers17152464 (PMC12346087; doi:10.3390/cancers17152464)
Supplement: Supplementary file 1 [file cancers-17-02464-s001.zip › cancers-3747472-supplementary.pdf]

Supplementary Materials:

Supplementary Table S1. Neuropeptide List

Gene Name	Gene Symbol
Pituitary adenylcyclase-activated peptide gene	**ADCYAP1**
Adiponectin gene	**ADIPOQ**
Adrenomedullin gene	**ADM**
Adrenomedullin-2 gene	**ADM2**
Agouti-related protein homolog gene	**AGRP**
Angiotensin gene	**AGT**
Apelin gene	**APLN**
Agouti-signaling protein	**ASIP**
Vasopressin gene	**AVP**
Calcitonin I gene	**CALCA**
Calcitonin II gene	**CALCB**
Cocaine- and amphetamine-regulated transcript gene	**CART**
Cocaine- and amphetamine-regulated transcript protein	**CARTPT**
Cerebellin-1 gene	**CBLN1**
Cerebellin-2 gene	**CBLN2**
Cerebellin-3 gene	**CBLN3**
Cerebellin-4 gene	**CBLN4**
Cholecystokinin gene	**CCK**
Glucagon gene	**CGC**
Chromogranin A gene	**CHGA**
Chromogranin B gene	**CHGB**
Corticotropin-releasing hormone gene	**CRH**
Cortistatin gene	**CST**
Diazepam-binding inhibitor	**DBI**
Endothelin 1 gene	**EDN1**
Endothelin 2 gene	**EDN2**
Endothelin 3 gene	**EDN3**
Galanin gene	**GAL**
Galanin-like peptide precursor gene	**GALP**
Gastrin gene	**GAST**
Growth hormone releasing hormone gene	**GHRH**
Ghrelin gene	**GHRL**
Gastric inhibitory peptide gene	**GIP**
Gonadotropin-releasing hormone gene	**GnRH1**
Gonadotropin-releasing hormone gene	**GnRH2**
Gastrin releasing peptide gene	**GRP**
Hypocretin gene	**HCRT**
Islet amyloid polypeptide gene	**IAPP**

IGF-1 gene	**IGF1**
IGF-2 gene	**IGF2**
Insulin gene	**INS**
Metastasis-suppressor KiSS	**KISS1**
Kininogen-1 gene	**KNG1**
Leptin/ob gene	**LEP**
Ly-6/neurotoxin-like protein 1	**LYNX1**
Motilin gene	**MLN**
Neuromedin B gene	**NMB**
Neuromedin S gene	**NMS**
Neuromedin U gene	**NMU**
Neuropeptide B gene	**NPB**
Neuropeptide FF gene	**NPFF**
Atrial natriuretic factor gene	**NPPA**
Brain natriuretic factor gene	**NPPB**
Natriuretic peptide precursor C gene	**NPPC**
Neuropeptides S gene	**NPS**
Neuropeptide W gene	**NPW**
Neuropeptide Y gene	**NPY**
Neurotensin gene	**NTS**
Nucleobindin-2/NEFA gene	**NUCB2**
Neurexophilin-1	**NXPH1**
Neurexophilin-2	**NXPH2**
Neurexophilin-3	**NXPH3**
Neurexophilin-4	**NXPH4**
Oxytocin gene	**OXT**
Visfatin gene	**PBEF1**
Pro-dynorphin gene	**PDYN**
Pro-enkephalin gene	**PENK**
Melanin-concentrating hormone gene	**PMCH**
Orphanin gene, pre-pronociceptin gene	**PNOC**
Pro-opiomelanocortin gene	**POMC**
Pancreatic polypeptide gene	**PPY**
Prolactin	**PRL**
Prolactin-releasing peptide	**PRLH**
Parathyroid hormone-like hormone gene	**PTHLH**
Peptide YY gene	**PYY**
Resistin-like alpha gene	**RETLA**
Resistin-like beta gene	**RETLB**
Resistin-like ggammane	**RETLG**
Resistin gene	**RETN**
Gonadotropin inhibitory hormone gene, RF-amide related peptide gene	**RFRP**
Relaxin-1 gene	**RLN1**
Relaxin-2 gene	**RLN2**

Relaxin-3 gene	**RLN3**
Secretogranin II gene	**SCG3**
Secretin gene	**SCT**
Secretory granule neuroendocrine protein 1, 7B2 gene	**SGNE1**
Somastostatin gene	**SST**
Preprotachykinin A gene	**TAC1**
Preprotachykinin B gene	**TAC3**
Tachykinin-4	**TAC4**
Thyrotropin-releasing hormone gene	**TRH**
Ubiquitin-like 5	**UBL5**
Urocortin gene	**UCN**
Urocortin II gene	**UCN2**
Urocortin III gene	**UCN3**
Urotensin-II	**UTS2**
Urotensin-II domain containing	**UTS2D**
VGF nerve growth factor inducible protein	**VGF**
Vasoactive intestinal peptide gene	**VIP**

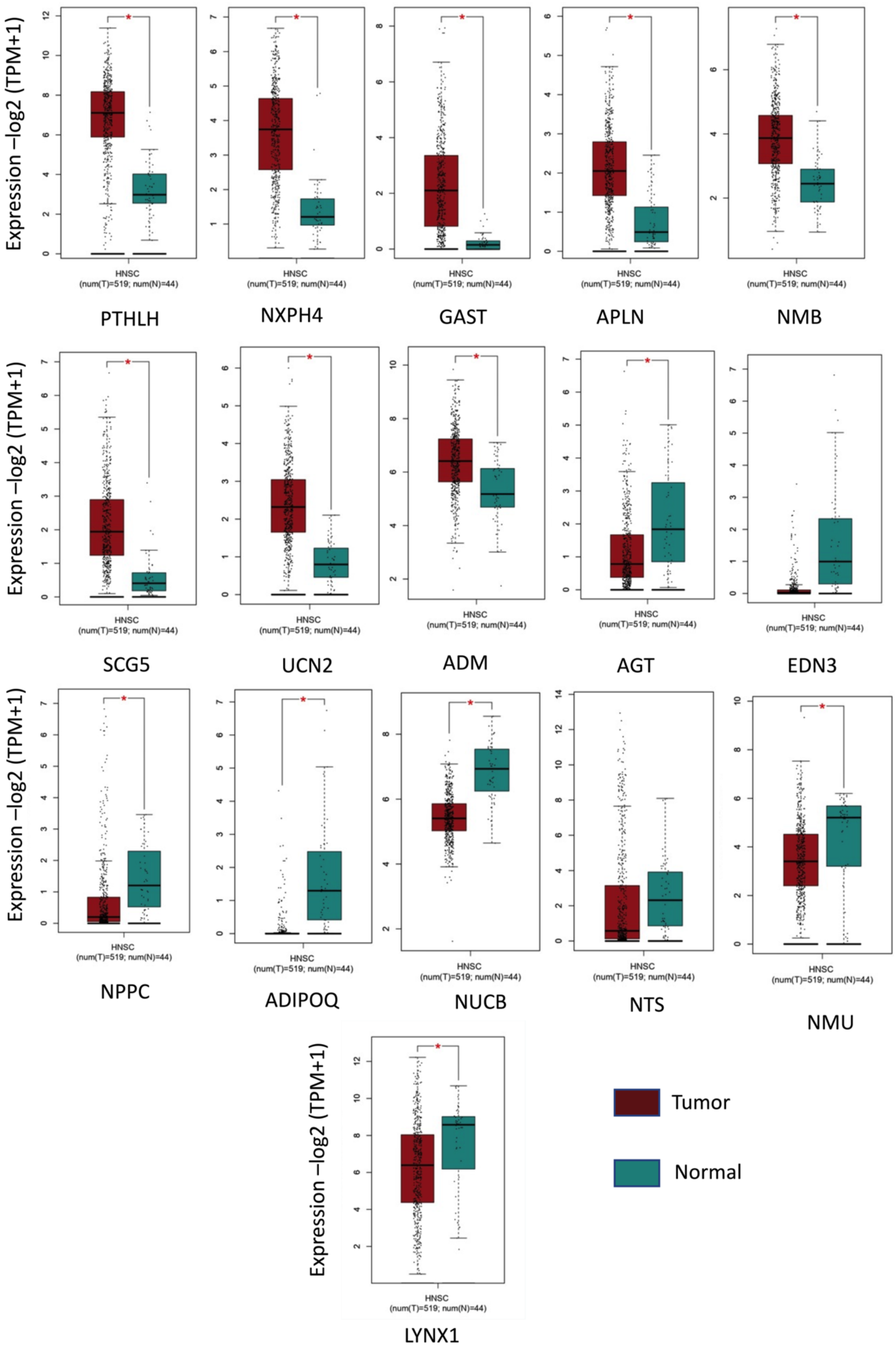

Supplementary Figure S1: The individual mRNA expression boxplot of the upregulated and downregulated neuropeptide genes (having log2 fold change >=1), in HNSC and normal tissue in GPIA2 (http://gepia2.cancer-pku.cn/#analysis accessed on 10 March 2025; *p < 0.05).

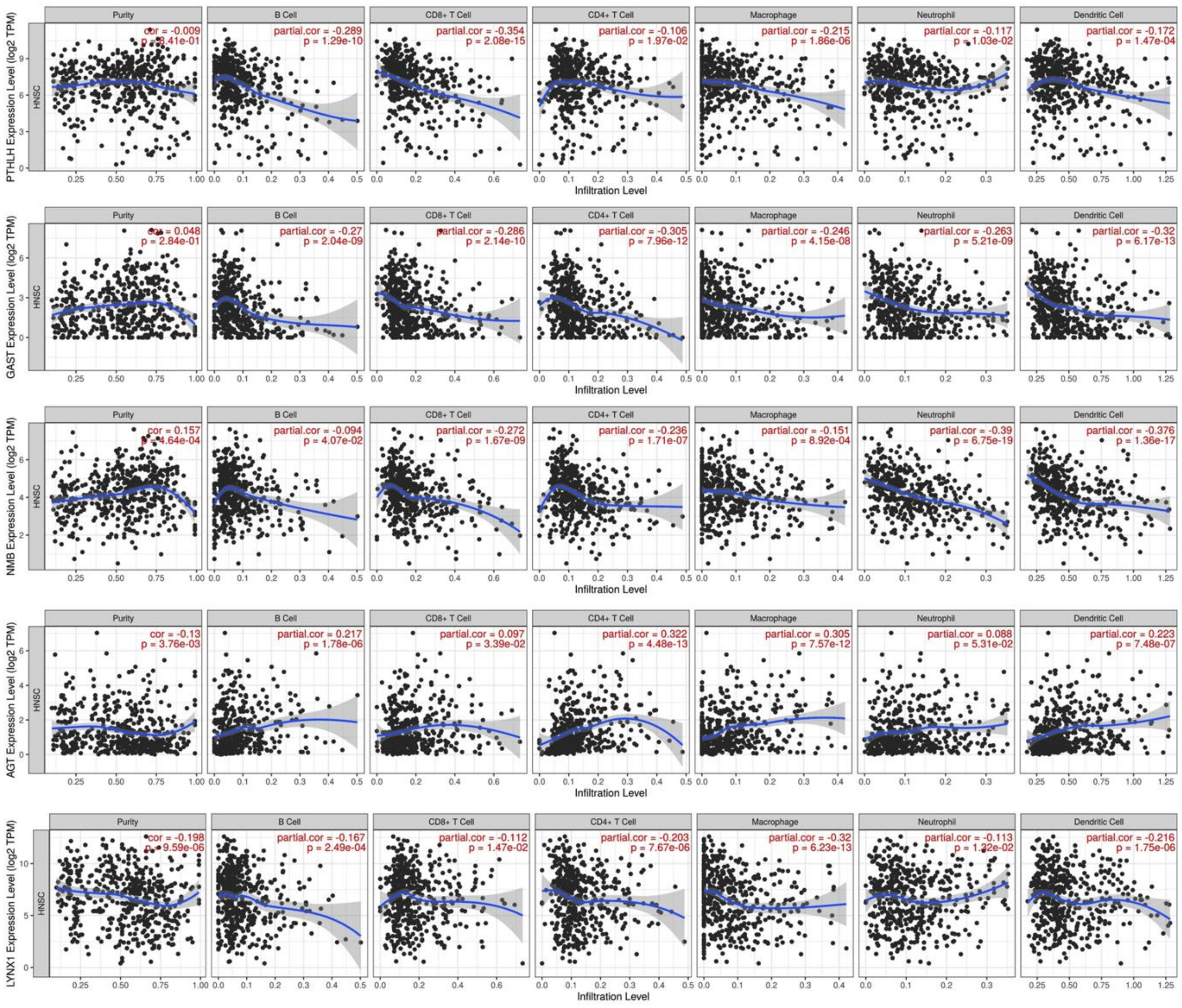

Supplementary Figure S2: Correlation of immune cells infiltration level of the selected neuropeptides which also showed significant differential expression: PTHLH, GAST, NMB, AGT and LYNX1 (https://cistrome.shinyapps.io/timer/ accessed on 14 March 2025).

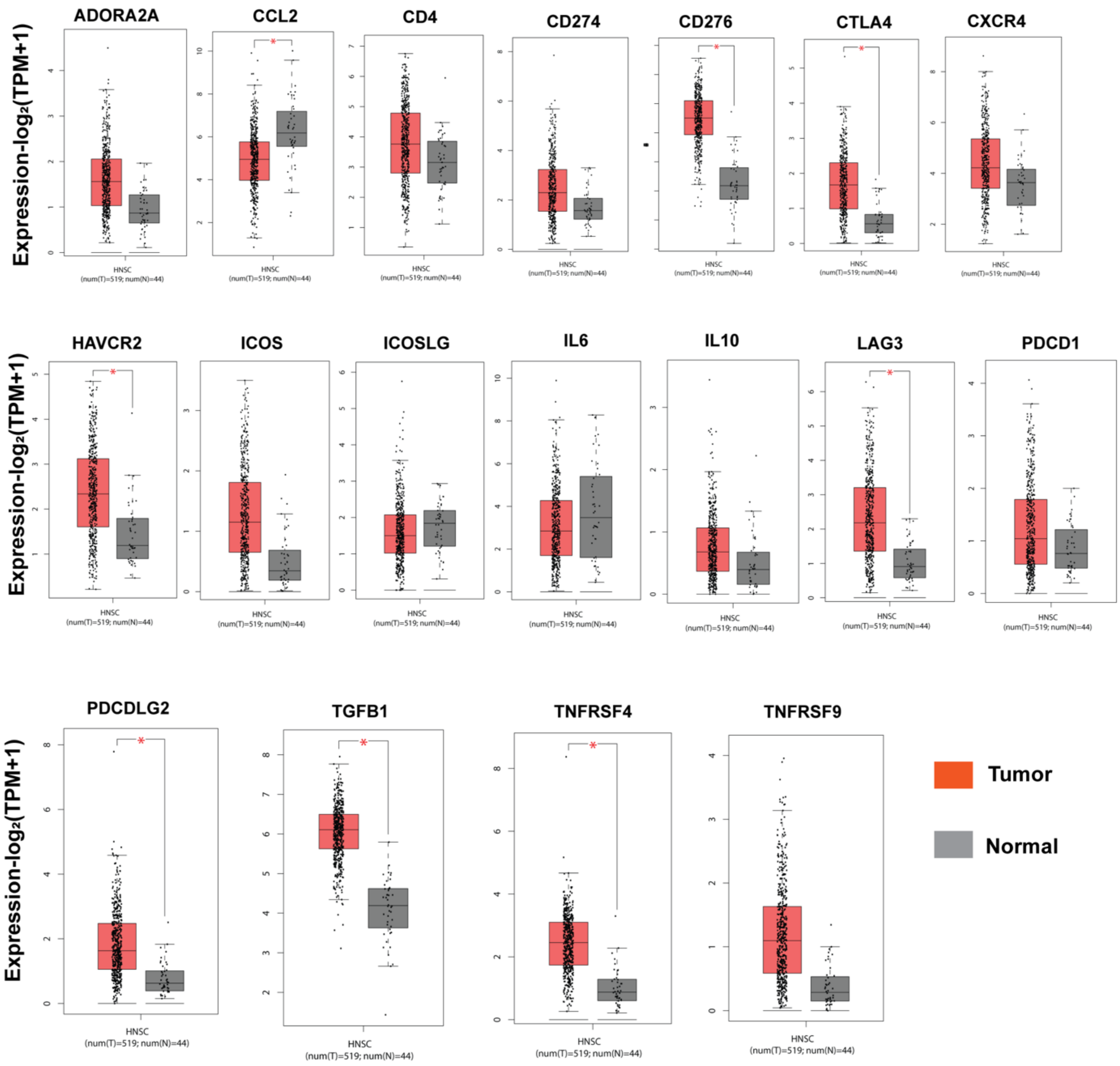

Supplementary Figure S3 Expression of immune checkpoint genes (ICGs) in HNSC (*$p<0.05$). CD276, CTLA4, HAVCR2, LAG3, PDCDLG2, TGFB1 and TNFRSF4 showed significant upregulation in HNSC tumor as compared to normal tissue; while CCL2 was significantly downregulated.

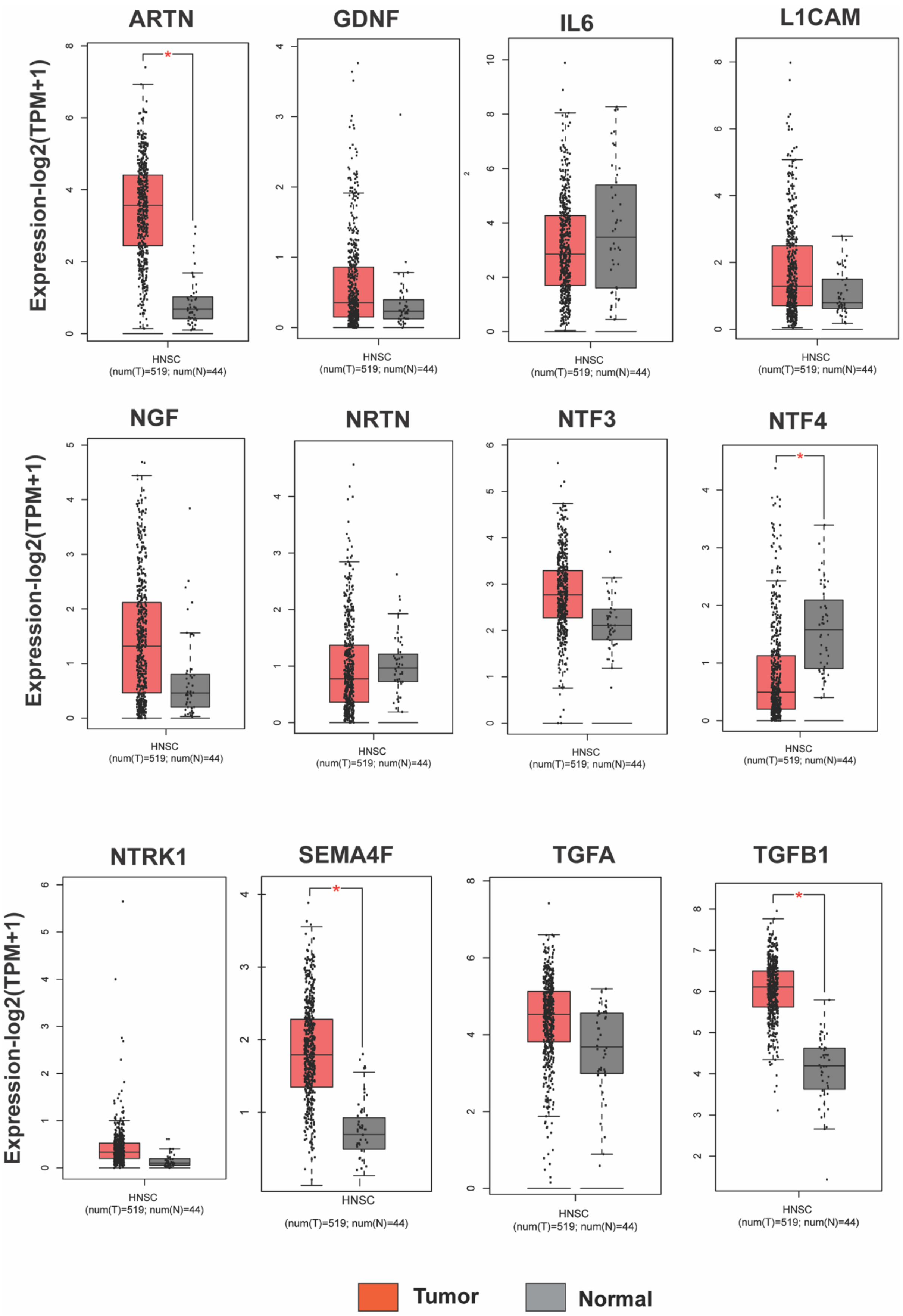
ARTN
GDNF
IL6
L1CAM
NGF
NRTN
NTF3
NTF4
NTRK1
SEMA4F
TGFA
TGFB1
Expression-log2(TPM+1)
HNSC
(num(T)=519; num(N)=44)
Tumor
Normal

Supplementary Figure S4 Expression of neurotrophic factors/ neurotrophins in HNSC (*p<0.05). In HNSC, neurotrophins gene-ARTN, SEMA4F and TGFB1 were significantly upregulated as compared to normal counterparts.
